# Effect of Iron/Folic Acid Supplementation on the Outcome of Malaria Episodes Treated with Sulfadoxine-Pyrimethamine

**DOI:** 10.1155/2014/625905

**Published:** 2014-01-19

**Authors:** Sunil Sazawal, Robert E. Black, Ibrahim Kabole, Arup Dutta, Usha Dhingra, Mahdi Ramsan

**Affiliations:** ^1^Department of International Health, Johns Hopkins Bloomberg School of Public Health, 615 North Wolfe Street, Room E8527, Baltimore, MD 21205, USA; ^2^Center for Public Health Kinetics, New Delhi, India; ^3^Public Health Laboratory-Ivo de Carneri, Wawi, Chake-Chake, Pemba, Zanzibar, Tanzania

## Abstract

Folic acid supplementation may potentially alter the efficacy of sulfadoxine-pyrimethamine (SP) treatment in children with malaria. However, there is lack of evidence from randomized controlled trials and effects of folic acid supplementation on clinical efficacy of SP therapy remain moderately understood among children. In a double masked, placebo-controlled trial among preschool children in Pemba Island (Tanzania), iron and folic acid supplementation (Fe/FA) showed an increased risk of hospitalizations and death. In the present paper, we evaluated if folic acid supplementation reduced the efficacy of malaria treatment and thereby contributed to observed adverse effects. During the study, 1648 children had confirmed malarial episodes and received either sulphadoxine-pyrimethamine (SP) treatment and iron folic acid or SP treatment and placebo. These children were evaluated for recovery and incidence of hospitalization during the next 15, 30, and 140 days. Two groups did not differ in malarial episode or hospitalization rate on subsequent 15, 30, and 140 days. Altered efficacy of SP by folic acid was not observed and did not contribute to adverse events in the previous trial. This trial is registered with Controlled-trials.com ISRCTN59549825.

## 1. Introduction

Malaria affects approximately 219 million people each year (range 154–289 million) with an estimated 660,000 deaths. Ninety percent of malaria deaths occur in Africa, where malaria accounts for about one in six of all childhood deaths (http://www.unicef.org/health/index_malaria.html). With widespread emergence of chloroquine resistant *P. falciparum* infections, drugs targeting the critically important folate metabolism of malarial parasite have been frequently used [1]. In a double masked, placebo-controlled trial among preschool children in Pemba island (Tanzania), iron and folic acid supplementation (Fe/FA) based on earlier WHO guidelines [2] showed an increased risk of hospitalizations and death [3]. Children during this study were receiving sulfadoxine-pyrimethamine (SP), an antifolate antimalarial drug as a first-line treatment for malaria. Sulfadoxine is known to act by inhibition of dihydropteroate synthetase while pyrimethamine competitively inhibits dihydrofolate reductase thereby blocking the endogenous pathway whereby plasmodium parasites produce folate de novo. In addition, it blocks uptake of and/or utilization by malaria parasites of exogenous folic acid that may transiently occur in circulation following ingestion of high supplemental doses. It has been suggested that FA supplementation (at least in higher doses) during SP treatment could adversely affect the inhibitory effect of SP on parasite growth, by providing folate to the parasite [4]. However, there is lack of evidence from randomized controlled trials and effects of FA supplementation on clinical efficacy of SP therapy among children remain moderately understood.

The present study investigates recovery and rehospitalization rate among 1648 laboratory confirmed malaria cases treated with SP with and without FA supplementation to evaluate if the observed increase in adverse events and mortality could be attributable to FA supplementation affecting the efficacy of malaria treatment.

## 2. Materials and Methods

The details methods of the study have been reported elsewhere [3]. In the parent study which contributed the sample for this study, after taking consent from the parents, children (aged 1 to 35 months) were enrolled and randomly allocated to receive one of the four preparations: (a) iron (Fe) (12.5 mg/day) and folic acid (FA) (50 *μ*g/day), or (b) Fe, FA and zinc (Zn) (10 mg/day), or (c) Zn alone or (d) placebo. Children <12 months received half the dose. All the children also got vitamin A as per WHO recommendations. They were visited weekly at home when the supplement was delivered and information on mortality and hospitalization was collected. All hospitalizations were prospectively monitored, and blood samples were collected.

In the substudy, 3171 children in the age group of 1–35 months were included as a part of which a detailed physical examination was performed; data related to their height and weight was taken and parental interview was conducted by clinicians and trained health workers. A 3 mL venous blood sample was also obtained from children for detailed haematological analysis (using portable hemoglobinometer-HemoCue, AB, Angelholm, Sweden), erythrocyte zinc protoporphyrin (using hematofluorometer—Aviv Biochemical, Lakewood, NJ), and malarial parasite count (thick and air-dried thin films were prepared and stained with Giemsa and read by light microscope with a ×100 oil immersion lens). Repeat assessments were performed at 6 months and 12 months after enrollment. Hospitalizations of the children in any of the five hospitals of Pemba were documented; information included details on illnesses of admitted children, tests for malaria parasites, discharge diagnosis and treatment received, or death. As per the WHO recommendations, for treatment purpose, cases were defined as all confirmed malaria with parasite count of ≥125 per 200 leucocytes. In these children and children visiting hospitals, a dose of sulfadoxine-pyrimethamine (SP) as a first-line treatment for malaria was provided by the study staff after laboratory confirmed diagnosis of malaria. A study worker read the consent statement to the primary caregiver and signed the form if consent was given. The trial was approved by the Institutional Review Board (IRB) at Johns Hopkins University and the Zanzibar Research Council.

For the present study, follow-up morbidity and subsequent 140-day hospitalization data for all 1648 children who were identified as malaria positive by research laboratory and given SP treatment was extracted.

Analysis compared children receiving SP and placebo with children receiving SP and Fe/FA or Fe/FA/Zn. Further to assess the effect of folic acid, we combined the data from Fe/FA and Fe/FA/Zn (called FA group). We estimated the relative risk (RR and 95% confidence intervals (95% CI)) of duration of treated episode of malaria extending beyond 4 days or 7 days and the RR (95% CI) of hospitalization during consequent 15 days, 30 days, or 140 days.

## 3. Results

**Figure 1:**
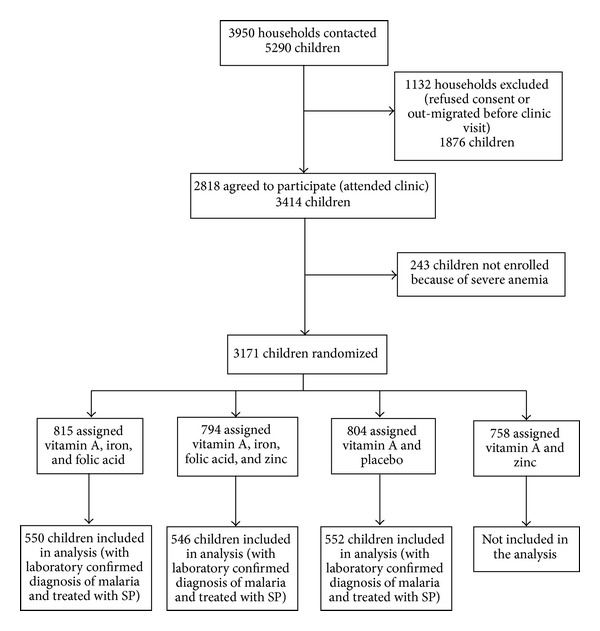
Study participants flow diagram.

For this analysis, out of the 4 groups in the previous trial, we included data from 3 groups with laboratory confirmed malaria: 552 children in placebo group, 550 children in Fe/FA group, and 546 children in Fe/FA/Zn group and compared children receiving SP and placebo with children receiving SP and Fe/FA or Fe/FA/Zn. In all there were 1096 children who received FA along with SP (FA group).  Figure 1 shows the study participants flow diagram. The relative risk of treated malarial episode extending beyond 4 days or beyond 7 days among those receiving FA as compared to the placebo was 1.03 (95% CI 0.99–1.07) and 0.99 (95% CI 0.93–1.09), respectively. Risk of hospitalization in the FA group during subsequent 15, 30, and 140 days was 27% (RR: 0.73; 95% CI: 0.38–1.45), 15% (RR: 0.85; 95% CI: 0.49–1.49), and 5% (RR: 0.95; 95% CI: 0.69–1.30) lower than the placebo group. However, these differences were not statistically significant (Table 1).

## 4. Discussion

Our results suggest that consumption of FA during the SP treatment for confirmed uncomplicated malaria did not increase the risk of prolonging the duration of treated malarial episode nor did it result in relapse of illness with higher hospitalization rates during subsequent 15, 30, or 140 days. Although this analysis cannot definitively address the question of effect of FA supplement on efficacy of SP, they do exclude this effect being responsible for the observed increase in adverse events associated with Fe/FA supplementation in the previous trial [3].

Biosynthesis of folate in the malarial parasite and the folate dependent reactions that it can carry out are not fully defined [1]. There are two pathways for the acquisition of folates: de novo biosynthesis and salvage from the host plasma in vivo. Existence of the de novo folate synthetic pathway is supported as *P. falciparum* grown in culture converts Guanosine-5′-triphosphate to polyglutamated derivatives, mainly methyltetrahydrofolate pentaglutamate [5, 6]. Plasmodia were long regarded as being unable to utilise intact exogenous folate (the salvage pathway) for tetrahydrofolate formation. However, evidence for synthesis of polyglutamated end products from folate provided direct evidence that folate salvage occurred in malarial parasites and that tetrahydrofolate could be acquired by salvage mechanisms as well [6]. However “de novo” synthesis appears more efficient than the utilization of salvaged folate [1].

In the red cell, methyltetrahydrofolate exists highly concentrated almost exclusively as polyglutamate. It has been suggested that plasmodium cannot use this large store of reduced folate because of lack of the enzyme glutamylhydrolase to remove the polyglutamate moieties [7]. Clinically, there is a strong correlation between sulfadoxine-resistant forms of the parasite carrying mutations in the dihydropteroate synthetase gene and the usage of sulfadoxine, suggesting that in case of infections in the human host, folate salvage cannot completely satisfy the parasite's requirements [1].

Sulfadoxine is a competitive inhibitor of the malarial enzyme dihydropteroate synthetase required for the condensation of p-aminobenzoate (pABA) and pterin in the de novo synthesis of folate. The use of sulfadoxine alone in *P. falciparum* malaria is complicated by the ability of many strains to use the exogenous folate present in the host, thus obviating the need for de novo synthesis and bypassing sulfadoxine inhibition of dihydropteroate synthetase. Pyrimethamine selectively inhibits the plasmodial enzyme dihydrofolate reductase to markedly different extent depending on its source, with the plasmodial enzyme inhibited by a concentration 3500 times less than the human enzyme [8]. In strains of *P. falciparum* that use exogenous folate efficiently, sulfadoxine inhibition can be restored by pyrimethamine. Thus, the combination of sulfadoxine and pyrimethamine (SP) acts synergistically, inhibiting two key enzymes in the biosynthesis and utilization of folate in *P. falciparum* and thus the synthesis of DNA and cell growth [9].

In an earlier study, Tong et al. provided 5 *μ*g FA daily for 12 days along with SP therapy to adults and reported no difference in parasite clearance [10]. However, more recent studies among African children receiving 10 *μ*g of FA along with standard SP therapy indicate that folic acid supplementation was associated with increased survivability of parasites beyond 28 days—a parasitological failure [11]. Similar results were recently reported from Zambia among 183 children with severe malaria, given dose of 1 mg/d for 14 days [12]. Nonserious adverse events and other clinical features were comparable in the two study groups. Similarly, in a study among 191 Malawian children with *P. falciparum* infection, a high blood folate concentration was associated with a 1.5-fold increased risk of late treatment failure [13].

In spite of the observed parasitological failure, studies that have evaluated both parasitological failure and clinical failure, have not reported any significant difference in clinical failure of therapy or adverse clinical outcomes [4, 14].

Our study was not designed to evaluate the association between the different folate doses and parasite density or clearance of malarial parasites, and this paper does not address that outcome. The supplement formulation (specific amounts of each nutrient) used for the study was the one which is recommended by Tanzania Food and Nutrition Center with the assumption that it can later be adopted for a national nutritional program. However, we did not find any evidence of reduced efficacy of SP therapy by folic acid (50 *μ*g/day) in regard to clinical recovery, relapse, or severity during 15 or 30 days after SP treatment. Therefore, it seems unlikely that folic acid supplement could have been responsible for higher rate of adverse events associated with Fe/FA supplementation in the previous trial at least by a mechanism affecting the efficacy of SP treatment.

The present study indicates that the dose of folic acid used had no effect on SP efficacy and therefore suggests that the negative impact on morbidity observed could not have been due to folic acid affecting antimalarial treatment. The evidence for the need of supplementing folic acid is weak and in areas with high malaria burden and SP being used as first-line treatment withholding folic acid may be advisable.

## Figures and Tables

**Table 1:** Hospitalizations at 15 days, 30 days, and 140 days following SP treatment.

Groups	*N*	Day 15		Day 30		Day 140	
SP and		*n*	RR (95% CI)	*n*	RR (95% CI)	*n*	RR (95% CI)
Placebo	552	17	—	23	—	63	—
Folic acid	1096	25	0.73 (0.38–1.45)	39	0.85 (0.49–1.49)	119	0.95 (0.69–1.30)
Fe/FA	550	14	0.82 (0.38–1.77)	19	0.82 (0.42–1.58)	62	0.99 (0.68–1.42)
Fe/FA/Zn	546	11	0.65 (0.27–1.46)	20	0.87 (0.46–1.67)	57	0.90 (0.62–1.32)

SP: sulfadoxine and pyrimethamine; Fe: iron; FA: folic acid; Zn: zinc.

Folic acid = Fe/FA and Fe/FA/Zn groups.
